# Perceptions et utilisation de la Couverture Maladie Universelle (Plan Sésame) par les personnes âgées à Dakar (Sénégal), impacts sur les dépenses de santé liées au diabète et à l'hypertension

**DOI:** 10.48327/mtsi.v3i3.2023.320

**Published:** 2023-08-19

**Authors:** Bernard TAVERNE, Gabriele LABORDE-BALEN, Bintou RASSOUL TOP, Khoudia SOW, Mamadou COUMÉ

**Affiliations:** 1 TransVIHMI (Université de Montpellier, INSERM, Institut de recherche pour le développement), 911 avenue Agropolis, BP 64501, 34394 Montpellier Cedex 5, France; 2 Centre régional de recherche et de formation à la prise en charge clinique de Fann (CRCF), CNHU de Fann, Dakar, Sénégal; 3 Service de gériatrie, CNHU de Fann, Faculté de médecine de l'Université Cheikh Anta Diop de Dakar, Sénégal

**Keywords:** Personnes âgées, Hypertension artérielle, Diabète, Couverture Maladie Universelle (CMU), Sénégal, Afrique subsaharienne, Elderly, Hypertension, Diabetes, Universal health coverage (UHC), Senegal, Sub-Saharan Africa

## Abstract

**Introduction/justification:**

En 2006, l'État sénégalais a mis en place un programme de couverture maladie pour les personnes âgées ≥ 60 ans - le Plan Sésame - devant permettre de fournir une prise en charge médicale gratuite dans toutes les structures sanitaires publiques du pays. Ce dispositif a été intégré dans celui de la Couverture Maladie Universelle promue à partir de 2013. L'objectif de l'étude était de décrire et analyser l'utilisation des dispositifs de couverture en santé en 2020, par les personnes âgées, d'évaluer le montant des dépenses médicales engagées à l'occasion d'une consultation médicale pour le suivi de leur maladie (hypertension artérielle et diabète), et de calculer les restes à charge liés à la consultation.

**Matériel et méthodes:**

Enquêtes réalisées entre juillet 2020 et octobre 2021, dans deux structures sanitaires publiques dakaroises. Approche mixte : 1/ étude qualitative auprès de 35 personnes sélectionnées selon une procédure de choix raisonné : 23 personnes âgées (dont 12 femmes), et 12 acteurs de santé; 2/ étude quantitative transversale par questionnaire auprès de 225 personnes (dont 141 femmes) âgées ≥ 60 ans. Nous avons calculé le coût total de la consultation et des prescriptions associées ainsi que le reste à charge des patients. Il s'agit d'une étude exploratoire descriptive auprès d'un échantillon non représentatif de la population des personnes âgées du Sénégal.

**Résultats:**

L'enquête met en évidence l'hétérogénéité des connaissances des personnes âgées sur la couverture sanitaire, alors que le Plan Sésame est en place depuis plus de 10 ans. Les personnes les mieux insérées socialement sont celles qui savent le mieux utiliser les services de la CMU. Les professionnels de santé adhèrent au principe de la couverture maladie, mais la plupart ont des connaissances limitées sur le dispositif. La mise en œuvre du Plan Sésame apparaît variable selon les structures sanitaires; la couverture fournie par le Plan Sésame n'est que partielle : entre 30 et 50% des coûts médicaux; le reste à charge lié à une consultation pour les patients âgés présentant une HTA et/ou un diabète varie entre 24 000 et 28 000 XOF (francs CFA).

**Conclusion:**

En 2021 le Plan Sésame ne permet pas encore une totale gratuité des soins pour les personnes âgées, cependant son application se traduit tout de même par une réelle baisse des dépenses de santé des personnes âgées. Ces observations confortent la nécessité de travailler à la réduction du prix des prestations médicales et au renforcement de la CMU, afin d'améliorer l'équité et la performance du dispositif.

## Abstract (en)

**Introduction/rationale:**

In 2006, the Senegalese government set up a health coverage programme for people aged 60 and over - the Plan Sésame - to provide free medical care in all the country's public health facilities. This scheme has been integrated into the Universal Health Coverage (CMU) promoted from 2013. The objective of the study was to describe and analyse the knowledge and representations of professionals and users about health coverage and the Plan Sésame, the use of the scheme by the elderly, to evaluate the amount of medical expenses incurred during a routine medical consultation for the monitoring of their illness (hypertension and diabetes), and to calculate the out-of-pocket expenses related to the consultation.

**Material and methods:**

Study conducted between July 2020 and October 2021 in two public health facilities in Dakar. Mixed approach: 1/ qualitative study by semi-directive interviews, informal interviews, observations and field diary with 35 people selected according to a reasoned choice procedure with the aim of diversifying gender, age, social status, therapeutic itineraries for 23 people (including 12 women, ages between 60 and 85 years), and professional activities for 12 health actors; 2/ quantitative cross-sectional study by questionnaire of 225 people (including 141 women) aged 60 and over; we calculated the total cost of the consultation and associated prescriptions (complementary examinations and medicines) as well as the remaining medical expenses (out-of-pocket) and the cost of transporting patients. This is a descriptive exploratory study of a non-representative sample of the elderly population in Senegal.

**Results:**

The health professionals interviewed supported the principle of health coverage, but most of them had limited and sometimes imprecise knowledge of the existing schemes and the methods of access or the services covered. Their point of view about the consequences of the Plan Sésame on their practice reveals some contradictions: some complain about the increase in workload, the criticism is extended to all the free schemes which would have a negative impact on daily practice because of the increase in the number of consultations which would be linked to abuse by patients.

The interviews highlight the heterogeneity of the knowledge of elderly people about the health coverage intended for them, even though the Plan Sésame has been in place for over ten years. The interviews clearly show that the use of the health coverage system by the elderly depends closely on the information they have and their ability to use it, both for women and men. There is a close link between the level of social integration of people and their use of health coverage: the most socially integrated people are those who know how to use CMU services best. The use of health coverage by the elderly appears to vary according to the individual.

Although Plan Sésame is defined as part of a national strategy, its implementation varies according to the health structures and the periods; in the two study sites, the range of services covered by Plan Sésame is very limited, so the coverage provided by Plan Sésame is only partial: between 30 and 50% of the medical costs; the remaining cost of a consultation for elderly patients with hypertension and/or diabetes varies between 24,000 and 28,000 CFA francs.

These amounts must be put into perspective with the resources available to people. Statistical studies published in 2021 report that in Senegal the average daily expenditure is 1,390 CFA francs/person/day; and that almost 38% of the population lives on 913 CFA francs/person/ day, which is the poverty line calculated in 2019. Thus, the average out-of-pocket expenses for a follow-up consultation for hypertension, diabetes or a combination of the two diseases represent 15 to 30 days of daily expenditure. While the vast majority of elderly people in Senegal do not have a retirement pension, health expenses are therefore borne by their relatives. Within households, medical expenditure for the elderly competes with basic needs, particularly food, which usually take up more than half of household resources. This indispensable family support places the elderly in a situation of total dependence.

**Conclusions:**

In 2021, Plan Sésame does not yet allow for completely free care for the elderly. However, its application, even partial, has resulted in a real reduction in health care costs for the elderly. Its use remains limited due to inconsistent application by most health structures. Its impact is insufficient in view of the amounts that users have to pay in a context of social and economic vulnerability. These observations reinforce the need to work on reducing the price of medical services and strengthening the UHC, in order to improve the equity and performance of the system, and to make it fully functional in all health structures.

## Introduction

Depuis 2010, un consensus international s'est instauré en faveur d'une couverture sanitaire universelle (CSU) pour les pays à revenus faibles et moyens [23]. Pour les pays du Sud et notamment l'Afrique subsaharienne, cette orientation est prise dans le contexte particulier de la transition démographique et épidémiologique qui se traduit par le vieillissement de la population et l'augmentation du nombre des personnes âgées [27], et par l'accroissement de la prévalence des maladies non transmissibles (hypertension artérielle, diabète, cancer…) [10], sur fond de précarité socio-économique persistante des ménages, aggravée en 2020 par la pandémie de Covid-19 et les mesures sanitaires associées [21, 29].

La généralisation de la couverture sanitaire universelle doit permettre de favoriser l'accès aux soins tout en réduisant les inégalités de santé en « ne laissant personne derrière » [13, 24]. Cependant, en 2023, dans la plupart des pays d'Afrique, encore très peu de personnes âgées bénéficient d'un dispositif de couverture sanitaire [26]. En 2013, l'État sénégalais a mis en place un dispositif de couverture sanitaire nommé Couverture Maladie Universelle (CMU) visant à fournir une prise en charge - partielle ou totale - des frais de santé pour l'ensemble de la population, ainsi qu'un programme de Bourse de sécurité familiale (BSF) consistant en l'attribution d'une bourse trimestrielle de 25 000 XOF (38 €) accordée à titre individuel sur critères de pauvreté [7, 9], pour une durée de 5 ans, ouvrant un droit à une inscription gratuite dans les mutuelles de santé communautaires. La CMU est principalement basée sur l'affiliation volontaire à des mutuelles de santé communautaires. Elle a également regroupé différents mécanismes de couverture préexistants, notamment plusieurs initiatives de gratuité - parmi lesquelles la gratuité des soins pour les personnes âgées de 60 ans et plus, appelée « Plan Sésame ».

Plusieurs régimes de couverture médicale existent au Sénégal pour les personnes âgées. Les anciens salariés du secteur privé sont couverts par l'Institution de prévoyance retraite du Sénégal (IPRES) qui fournit des soins dans les Centres médico-sociaux de l'IPRES de Dakar et de 11 autres grandes villes. Les retraités de la fonction publique sont couverts par le Fonds national de retraite (FNR) à travers l'« Imputation budgétaire », dans la continuité de la couverture dont ils bénéficiaient avant leur retraite. Ces dispositifs concernent entre 10 et 30% des personnes âgées [2]. Le Plan Sésame a été instauré pour venir en complément de ces dispositifs et fournir une couverture médicale à l'ensemble des personnes âgées qui n'en bénéficiaient pas.

Le Plan Sésame a été initié en 2006. Il prévoyait de fournir une prise en charge médicale gratuite dans toutes les structures sanitaires publiques du pays (postes de santé, centres de santé, hôpitaux) aux personnes âgées de 60 ans et plus, ne bénéficiant pas d'autres dispositifs de couverture maladie, ou de manière complémentaire à celles bénéficiant de prises en charge partielles. Les structures de soins fournissent les prestations qui leur sont payées en retour par l'État. En pratique, la mise en œuvre du Plan s'est heurtée à plusieurs difficultés qui en ont très tôt limité la portée. En 2010, certaines prestations ont été exclues pour limiter l'accroissement des dépenses (médicaments de spécialités, prothèses, IRM, pacemakers…) [1]. Plusieurs études réalisées entre 2010 et 2013 ont décrit les causes de divers dysfonctionnements et leurs conséquences [14, 25]. À la création de la CMU, le financement du Plan Sésame y a été intégré et en 2014 le ministère de la Santé a transféré sa gestion à la Cellule d'appui à la CMU afin d'en rationaliser la gestion.

Dans le cadre du programme de recherche Unissahel [28], nous nous sommes intéressés à l'impact du dispositif de couverture maladie sur les dépenses de santé des personnes âgées à Dakar, en focalisant l'attention sur celles atteintes de diabète et/ou d'hypertension, maladies dont la prévalence est déjà très élevée au Sénégal [15, 17] et qui nécessitent un suivi et des soins réguliers. La régularité du suivi médical est en effet indispensable pour éviter les complications invalidantes ou la mort pour les personnes - infarctus cardiaque, accident vasculaire cérébral, cécité, etc. -, qui sont coûteuses pour la collectivité. Plus précisément, il s'agissait de décrire l'utilisation des dispositifs de couverture sanitaire par les personnes âgées, d'évaluer le montant des dépenses médicales engagées à l'occasion d'une consultation de routine pour le suivi de leur maladie et de calculer les restes à charge liés à cette consultation.

## Méthode

### Site d'étude et population

L'étude a été réalisée entre juillet 2020 et octobre 2021, dans deux structures sanitaires publiques dakaroises, le centre de santé Gaspard Kamara et le centre de diabétologie Marc Sankalé de l'hôpital Abass Ndao.

Le centre de santé Gaspard Kamara est situé dans le quartier Sicap-Amitié, dans la commune de Fann-Amitié-Point E de Dakar. Dans ce centre de santé de niveau 2 dans la pyramide sanitaire, l'offre de service est diversifiée : médecine générale, maternité et bloc chirurgical obstétrique, néonatologie, spécialités médicales (pédiatrie, gynéco-obstétrique, cardiologie, urologie, dermatologie, ophtalmologie, odontologie), laboratoire et imagerie médicale. L'accueil des urgences, maternité et bloc opératoire est assuré 24h/24h. L'offre de soins est proche de celle proposée dans les centres hospitaliers. Cette structure attire des patients résidant à proximité mais aussi venant de communes parfois éloignées.

Le centre de diabétologie Marc Sankalé est situé dans l'enceinte de l'hôpital Abas Ndao, dans le quartier Gueule Tapée de la commune d'arrondissement Gueule Tapée-Fass-Colobane de Dakar. Il s'agit du centre national de référence dans la prise en charge ambulatoire du diabète. Le recrutement de patients est principalement local, mais certains viennent de régions éloignées de Dakar et quelques-uns de pays voisins. Ces deux structures du secteur public sont situées en milieu urbain. Elles sont appréciées par la population pour la bonne qualité des soins qui y sont dispensés; nombre de patients choisissent de s'y rendre même s'ils résident dans des quartiers éloignés.

### Collecte des données, mode d'analyse et de calcul

Les enquêtes (qualitative et quantitative) ont été réalisées par une assistante de recherche sénégalaise titulaire d'un Master 2 en sociologie, ayant reçu une formation sur les aspects historiques et sociaux à propos des dispositifs de couverture sanitaire, et d'éthique de la recherche. Elle a été supervisée par trois anthropologues seniors.

L'enquête qualitative a mobilisé les méthodes usuelles (entretiens semi-directifs, entretiens informels, observations et journal de terrain) [22] auprès de 35 personnes. Les personnes ont été sélectionnées selon une procédure de choix raisonné dans un objectif de diversification des sexes, âges, statuts sociaux, itinéraires thérapeutiques pour les patients et activités professionnelles pour les acteurs de santé afin de prendre en compte la variabilité des points de vue sur les thèmes abordés. Les entretiens avec les professionnels de santé et les acteurs de la protection sociale abordaient leurs expériences et pratiques professionnelles quotidiennes, leur perception des dispositifs de couverture sanitaire et de leurs impacts. Les entretiens avec les personnes âgées concernaient des éléments biographiques, l'histoire de leur maladie et de leurs parcours thérapeutiques, les modalités de prise en charge de leurs dépenses de santé, les solidarités familiales et la place de la couverture sanitaire dans leurs dépenses de santé. Les entretiens ont été réalisés dans les deux formations sanitaires, dans les locaux de travail des structures de protection sociale, ou à domicile pour certains patients. Les entretiens conduits en français ou en wolof, d'une durée variable à la convenance des personnes, ont été enregistrés, puis traduits et retranscrits; les verbatims ont fait l'objet d'une analyse thématique de contenu.

L'enquête quantitative était proposée sur une base volontaire aux patients au décours de leur consultation. Tous les participants ont reçu une brève présentation de l'étude et ont eu l'occasion de poser des questions avant de donner leur consentement oral à participer. Les refus de participer à l'enquête ont été exceptionnels. Les informations ont été collectées à partir d'un questionnaire administré en face-à-face, au sortir d'une consultation médicale de routine. Ces consultations médicales de routine (ou de suivi) sont liées au réapprovisionnement en médicaments, avec une périodicité variable selon l'état clinique du patient. Elles ont lieu le plus souvent tous les trimestres. Une ou deux fois par an, elles sont l'occasion de réaliser des examens biologiques de suivi.

Les informations recueillies comprenaient : des données médico-sociales (âge, sexe, ressources économiques, pathologies et traitements habituels); des informations sur l'affiliation à un dispositif d'assurance maladie ou de gratuité; la nature des prestations médicales et leur coût au jour de la consultation; le montant des frais de déplacement aller-retour; l'existence de paiement informel et leur montant; les raisons d'un éventuel non-recours au dispositif de couverture sanitaire. Les dépenses médicales distinguaient : la consultation; les examens biologiques; l'imagerie médicale (radiographie, électrocardiogramme, échographie); les médicaments prescrits; les éventuelles autres dépenses médicales. Pour chaque type de dépense, le patient devait préciser qui avait finalement payé la dépense (lui-même, une structure de prise en charge, une association, une mutuelle ou une assurance).

Les données collectées ont permis de calculer le coût total de la consultation et le montant du reste à charge du patient. Le coût de la consultation médicale comprend les coûts liés à l'achat du ticket de consultation, aux ordonnances de médicaments, aux analyses biologiques et à l'imagerie médicale. Les frais de déplacement n'ont pas été comptabilisés dans le calcul du coût moyen médical, mais intégrés au montant du reste à charge. Le reste à charge est défini comme « la part de la dépense de santé que les ménages ont à payer directement lors des soins, après intervention de l'assurance maladie, de l'État ou des organismes d'assurance maladie complémentaire » [5]. Le calcul des coûts est basé sur les dépenses réelles engagées par le patient. Les coûts ont été mesurés en XOF (francs CFA) au moment de l'interview. Les montants enregistrés ont donné lieu à une analyse statistique descriptive élémentaire (moyenne, mini-maxi, médiane et IQ 1-3).

Les informations obtenues ont été confrontées et mises en perspective avec celles issues de la littérature concernant les données socio-démographiques du Sénégal, la CMU au Sénégal, ainsi que les coûts médicaux pour l'hypertension artérielle et le diabète au Sénégal et d'autres pays d'Afrique de l'Ouest.

### Considérations éthiques

Le protocole SEN18/20 « UNISSAHEL-Sénégal Couverture universelle en santé au Sahel » a été approuvé par le Comité national d'éthique pour la recherche en santé (CNERS) du Sénégal le 2 juillet 2018 sous le numéro 0000053/MSAS/DPRS/CNERS et le 18 juillet 2019 sous le numéro 0000118/MSAS/DPRS/CNERS.

### Participation communautaire et présentation des résultats aux acteurs

Le but de l'étude, sa conception et les instruments d'étude ont été étroitement conçus et développés par une équipe d'acteurs communautaires, de membres d'associations de patients et de chercheurs. Les résultats de la recherche ont été présentés avant publication aux membres du Conseil national des aînés du Sénégal (CNAS - la principale association de personnes âgées du pays), à l'Association sénégalaise de soutien et d'assistance aux diabétiques (ASSAD), aux responsables médicaux et administratifs des deux structures où ont été réalisées les enquêtes, et aux principaux acteurs de l'Agence nationale de la Couverture Maladie Universelle et du Ministère de la santé.

### Caractéristiques de la population

Les entretiens ont été réalisés avec 7 professionnels de santé (infirmiers, médecins) ayant entre 1 et 9 ans d'expérience professionnelle dans leur structure de travail; 5 agents administratifs et acteurs de la protection sociale (assistant social, caissier, « point focal mutuelle », « point focal Plan Sésame », trésorier de l'Association sénégalaise de soutien et d'assistance aux diabétiques-ASSAD) ayant entre 3 et 32 ans d'activité dans leur structure.

Les entretiens avec les personnes âgées ont concerné 23 personnes (12 femmes et 11 hommes, âges compris entre 60 et 85 ans). Parmi les femmes, 8 étaient veuves, 3 d'entre elles bénéficiaient d'une pension de réversion de leur époux versée par l'IPRES, 4 étaient bénéficiaires d'une Bourse de sécurité familiale, 1 seule femme recevait une pension de retraite en lien avec son emploi antérieur. Tous les hommes étaient mariés, 5 recevaient une pension de retraite, 1 était bénéficiaire d'une Bourse de sécurité familiale. 12 personnes étaient traitées pour un diabète (antidiabétique oral et/ou insuline), 3 pour diabète et HTA, 1 pour HTA seule, 7 personnes pour diverses affections (douleurs rhumatismales, troubles visuels, maladie bronchopulmonaire, pathologie urogénitale).

L'enquête quantitative a été menée auprès de 225 personnes (dont 141 femmes). La taille de l'échantillon a été définie a priori et par convenance, sans ambition de représentativité. L'âge moyen était de 68 ans pour les deux sexes, l'âge maximal de 96 ans; 81 personnes (36%) avaient 70 ans et plus (Tableau I). 20% des personnes déclaraient avoir encore une activité professionnelle (27% des hommes; 17% des femmes). Parmi les 24 femmes qui déclaraient avoir une activité professionnelle, 11 avaient plus de 65 ans. Parmi les 23 hommes qui déclaraient avoir une activité professionnelle, 15 avaient plus de 65 ans. 57 personnes (26%) bénéficiaient d'une pension de retraite, avec une répartition inégale selon le sexe : 37% des hommes ont déclaré bénéficier d'une pension de retraite contre 19% des femmes. Le niveau de ressources économiques mensuelles approximatif était inférieur à 25 000 XOF pour 27 personnes (12%), compris entre 25 000 et 50 000 XOF pour 51 personnes (23%), entre 50 000 et 100 000 XOF pour 68 personnes (30%) et supérieur à 100 000 XOF pour 79 personnes (35%).

**Tableau I:** Caractéristiques socio-économiques et proportion de bénéficiaires de la couverture maladie de la population d'enquête Socio-economic characteristics and proportion of the study population covered by health insurance

	Femme	Homme	Total
**N**	141	84	225
**Âge moyen (médian)**	68 (66)	68 (67)	68 (67)
**Âge max**	96	92	96
**Activité professionnelle permettant un revenu**			
**(n%)**			
**oui**	24 (17%)	23 (27%)	47 (20%)
**non**	117 (83%)	61 (73%)	178 (80%)
**Pension de retraite**			
**(n%)**			
**oui**	27 (20%)	31 (37%)	58 (26%)
**non**	114 (80%)	53 (63%)	167 (74%)
**Ressources mensuelles (quelle que soit la source)**			
**< 25 000 XOF**	25 (18%)	2 (2%)	27 (12%)
**25 - 50 000 XOF**	38 (27%)	13 (15%)	51 (23%)
**50 - 100 000 XOF**	41 (29%)	27 (32%)	68 (30%)
**≥ 100 000 XOF**	37 (26%)	42 (50%)	79 (35%)
**Bénéficiaire d'une couverture médicale (Plan S ± mutuelles, assurance)**	89 (63%)	60 (71%)	149 (66%)
**Ont fait appel à un soutien familial pour les dépenses de santé liées à la consultation**	94 (67%)	37 (44%)	131 (58%)
**Ont dû repousser la date de la consultation par incapacité financière**	10 (7%)	5 (6%)	15 (7%)

La répartition des personnes selon leur sexe et leur niveau de ressources révèle une forte différence entre les femmes et les hommes : 45% des femmes ont des ressources inférieures à 50 000 XOF/mois *vs* 20% des hommes; et ces derniers sont plus nombreux à avoir des ressources mensuelles supérieures à 100 000 XOF : 50% *vs* 26% des femmes. Les trois-quarts de ces personnes avaient un diabète, de l'hypertension, ou les deux maladies associées; les autres présentaient diverses affections ostéo-articulaires (arthrose et rhumatisme), infectieuses (« grippe »), ophtalmologiques, dermatologiques, etc.

## Résultats

### L'offre de service, une application variable du Plan Sésame

Bien que le Plan Sésame soit défini dans le cadre d'une stratégie nationale, sa mise en œuvre est variable selon les structures sanitaires et les époques. La décision imposée aux structures de santé de fournir gratuitement un ensemble de prestations et les délais de compensation par l'État ont privé ces structures d'une part de leurs ressources et créé des déséquilibres budgétaires dans leur gestion. La plupart ont réagi en restreignant l'offre de service; par exemple, aucune structure de santé ne fournit gratuitement les médicaments. Au moment de l'enquête, le centre Gaspard Kamara appliquait le Plan Sésame pour la prise en charge des seules consultations médicales réalisées par un médecin généraliste. Cette consultation, tarifée à 1000 XOF, était imputée au Plan Sésame; la consultation était ainsi gratuite pour le patient. Par contre, les consultations avec un médecin spécialiste (ophtalmologie, cardiologie, etc.) étaient facturées 5000 XOF et n'étaient pas couvertes par le Plan Sésame. L'ensemble des autres dépenses (achat de médicaments, examens biologiques et radio-logiques) n'était pas imputé au Plan Sésame par la structure.

Au centre Marc Sankalé, le Plan Sésame n'était plus en application depuis une année au moment de l'enquête (il a été rétabli en juin 2021). La consultation (2200 XOF) ainsi que toutes les autres prescriptions étaient donc à la charge complète de l'usager. Néanmoins, pour les examens biologiques et radiologiques, ces deux centres dirigeaient les patients vers des hôpitaux afin de leur permettre de bénéficier de la prise en charge par le Plan Sésame; ils évitaient ainsi de fournir eux-mêmes ces prestations, même lorsqu'elles étaient disponibles sur place. Par ailleurs, ces deux structures sanitaires ont établi des conventions avec des mutuelles de santé communautaires (12 pour le centre Gaspard Kamara [dont la Mutuelle Fann-Amitié-Point E], 5 pour le centre Marc Sankalé) et toutes deux acceptent la tarification liée à l'Imputation budgétaire/FNR.

### Le point de vue des professionnels et des usagers sur la couverture maladie et le Plan Sésame

#### Les professionnels de santé

Tous les professionnels de santé rencontrés affichaient une adhésion au principe de la couverture maladie, mais la plupart avaient des connaissances limitées et parfois imprécises sur les dispositifs existants, les modalités d'accès ou les prestations couvertes. « Nous, on fait les consultations. Pour savoir qui a accès, comment ça marche, il faut voir avec le service social » conseillait un infirmier. Un médecin reconnaissait : « Vous savez, quand on est médecin, on n'a pas trop besoin de ça [mutuelle]. Quand on a un membre de notre famille qui est malade, on va voir un collègue et il nous règle notre problème. » Peu ou mal informés sur les dispositifs en place, ils n'abordaient quasiment jamais ce sujet avec les patients lors des consultations.

La majorité des acteurs de santé déclaraient pourtant : « Le Plan Sésame est une bonne mesure car elle favorise l'accès aux soins pour les plus démunis. » Certains vantaient sa simplicité d'utilisation : « Pour le Plan Sésame, il suffit d'avoir 60 ans et une carte d'identité sénégalaise pour bénéficier de la gratuité » affirmait un médecin. D'autres mettaient en avant ses avantages : « On prend en charge la consultation, les bilans et les médicaments » disait un infirmier. Tous les acteurs de santé rencontrés affichaient une adhésion au principe moral de ce dispositif en faveur des personnes âgées. Globalement, ils affirmaient que la gratuité favorise l'accès aux soins des patients « qui ne sont pas solvables », les encourage à respecter les rendez-vous, permet un meilleur suivi médical et favorise la prise en charge des maladies chroniques, telles que l'hypertension et le diabète.

Mais dans le même temps, leur point de vue à propos des conséquences du Plan Sésame sur leur pratique s'avère plus nuancé et révèle quelques contradictions. Certains se plaignaient de l'augmentation de la charge de travail : « C'est bien beau, mais la charge de travail augmente pour nous médecins. Il faut aussi dire la vérité. On travaille beaucoup parce que les gens ne payent pas, ils viennent fréquemment, il faut être sincère et le dire » affirmait un médecin. Un infirmier précisait : « La charge de travail a augmenté, on a moins de temps pour les consultations. » La critique est élargie à l'ensemble des dispositifs de gratuité qui auraient ainsi un impact négatif sur la pratique quotidienne. À l'augmentation du nombre des consultations est associée la suspicion des abus de la part des patients : « Les gens ont tendance à abuser de tout ce qui est gratuit » selon un infirmier; une assistante sociale déplorait : « Les politiques de gratuité sont une très bonne initiative mais les gens en abusent. Avant, avec le Plan Sésame, les personnes âgées allaient se faire hospitaliser dans les structures chères, se disant que c'est l'État qui payait ! » Un gestionnaire se plaignait lui aussi de la charge de travail et des difficultés de trésorerie créées par le retard de la compensation versée par l'État : « C'est une bonne initiative, le problème majeur est le retard de remboursement, ce n'est pas possible de prendre en charge constamment les malades alors qu'on n'est pas remboursé à la fin. »

Les difficultés de trésorerie sont mises en avant pour expliquer les restrictions - voire la suppression - des prestations fournies (cf. supra) : « En fait, les médicaments ne font pas partie [de la prise en charge] pour le troisième âge, ce sont les parents qui vont acheter, ici seule la consultation est prise en compte » précisait la pharmacienne du centre Gaspard Kamara qui poursuivait : « La gratuité est très lourde par rapport à l'achat des médicaments, il faut qu'on renouvelle le stock alors que le remboursement ne suit pas directement, on est en train de donner des médicaments, mais le remboursement est très lent et il faut des médicaments. S'il n'y a pas de médicaments dans une structure ça ne marche pas et ce n'est pas bon, on peut tenir quelque temps, mais à la longue ce n'est pas bon. Pour la consultation il n'y a pas de problèmes parce que le personnel est là, mais pour les médicaments, c'est très difficile. »

La stratégie mise en place consiste à diriger les patients vers d'autres structures de soins « qui ont plus de budget pour faire les bilans gratuitement, comme l'hôpital Principal, l'hôpital Le Dantec, ou l'hôpital Fann. Ce n'est pas forcément dans une même structure qu'on peut faire toutes les prestations pour le Plan Sésame. Nous sommes dans un centre de santé du point de vue statut, certaines prises en charge sont référées au niveau des hôpitaux » précisait une assistante sociale.

Une « lettre de garantie », un « bulletin de référence » et l'ordonnance de prescription des examens sont remis aux patients. La « lettre de garantie » atteste l'inscription au Plan Sésame à partir de la structure sanitaire dont dépend le patient, le « bulletin de référence » atteste du respect de la pyramide sanitaire dans le parcours de soins. Muni de ces documents, le patient peut se rendre dans une structure de niveau sanitaire supérieur (un hôpital) où les frais liés aux examens seront couverts par le Plan Sésame. De cette manière, les structures périphériques transfèrent aux hôpitaux la charge financière qu'elles ne peuvent - ou ne souhaitent - pas assumer.

En pratique, cela complique et allonge le parcours de soins des patients qui ne sont pas certains de pouvoir bénéficier d'une prise en charge. En effet, les hôpitaux, confrontés aux mêmes difficultés liées au retard de versement de la compensation de l'État, instaurent eux aussi des restrictions comme le reconnaissait l'assistante sociale : « Il y a des hôpitaux qui ne prennent plus le Plan Sésame, par exemple l'hôpital Principal depuis 2 ans ne prend plus en charge les malades venant avec le Plan Sésame, ils prennent seulement les consultations, ils ne prennent plus les analyses et les radiographies parce que l'État ne leur a pas encore payé les prestations. » Tous les professionnels de santé sont informés de ces adaptations et savent bien que la gratuité des soins pour les personnes âgées est finalement partielle. Ces observations alimentent chez eux les discours sur les limites de la gratuité : « Quelle est la viabilité à long terme de tels dispositifs ? Tout ne peut pas être gratuit, c'est bien de mettre en place la gratuité mais je pense qu'il faudrait l'organiser un peu plus » remarquait un infirmier.

#### Les usagers

L'utilisation du dispositif de couverture sanitaire par les personnes âgées apparaît variable selon les individus. Les rares personnes qui perçoivent une pension de retraite, en tant qu'anciens salariés du secteur public ou privé, apparaissent les mieux informées sur les différents dispositifs : couverture par l'IPRES, l'Imputation budgétaire/FNR, et les mutuelles. Certains patients utilisent l'un ou l'autre de ces dispositifs selon les circonstances et le niveau de prise en charge, comme le rapportait un ancien fonctionnaire de ministère; il bénéficie de l'IB/FNR, il est adhérent à la mutuelle des fonctionnaires de l'État et aussi à la mutuelle de Gaspard Kamara [FAPE] : « J'utilise l'IPRES avec mon épouse, mais je viens faire des consultations ici et si j'ai des factures, soit je les dépose ici, soit à la mutuelle des fonctionnaires; làbas c'est plus pratique, donc je dépose là-bas mes remboursements et je suis remboursé à 50%, ici, je paye une partie, les 20%… y a pas mieux. »

Les entretiens avec les personnes âgées mettaient clairement en évidence que leur utilisation du dispositif de couverture maladie dépend étroitement de l'information dont elles disposent et de leur capacité à les utiliser, tant pour les femmes que les hommes. C'est notamment le cas de veuves bénéficiant de la couverture maladie de leur mari. L'une d'elles témoignait : « Je reçois une pension de mon mari [décédé depuis une dizaine d'années], on m'a dit que je pouvais bénéficier des soins à l'IPRES mais je n'y suis pas encore allée. » Une autre affirmait n'avoir jamais entendu parler de la possibilité de prise en charge par l'IPRES : « Je reçois une pension depuis que mon mari est décédé, c'est ma fille qui s'occupe de tout. » Les personnes âgées rencontrées étaient très peu informées de l'existence des mutuelles de santé communautaires, certaines affirmaient en avoir entendu parler mais n'en connaissaient pas le fonctionnement. Cette méconnaissance est observée également chez des personnes qui ont été affiliées à une mutuelle à travers le dispositif de Bourse de sécurité familiale : elles possèdent un carnet de mutuelle, mais ne savent pas précisément si l'exemption des paiements pour les consultations est liée à la mutuelle.

La connaissance par les personnes âgées de l'existence du Plan Sésame et son utilisation sont également variables. Certaines personnes âgées déclaraient ne pas le connaître, en avoir simplement entendu parler, ou affirmaient ne pas savoir comment en bénéficier alors même qu'elles avaient eu plusieurs contacts avec des structures de santé : « Je vais me renseigner; j'en ai entendu parler par un ami à qui on l'avait expliqué » (homme, 60 ans); « J'en ai entendu parler, mais je ne sais pas comment on peut faire pour l'avoir; chaque fois que je dois faire des analyses, je paye » (femme, 65 ans). Quelques rares personnes doutaient même de son existence : « J'en entends parler, mais je n'ai jamais vu ou entendu quelqu'un qui en a bénéficié » (femme, 66 ans). L'information sur l'existence du Plan Sésame apparaît variable selon les structures. L'orientation des personnes âgées vers le Plan Sésame est organisée de manière systématique dans certaines structures : « Lorsque je suis venue en consultation, le monsieur au guichet a regardé ma carte d'identité et m'a dit que je bénéficie du Plan Sésame et que je vais me faire consulter gratuitement » (femme, 65 ans); tandis que la possibilité d'une prise en charge par le Plan Sésame ne semble pas évoquée dans d'autres sites.

La majorité des personnes âgées rencontrées connaissent le Plan Sésame *via* la presse : « Je l'ai entendu à travers la radio; j'ai prêté attention car ils parlaient de maladies et de la possibilité de se faire soigner pour les personnes du 3^e^ âge » (homme, 63 ans); ou les rumeurs : « Le Plan Sésame, j'ai juste entendu dire que c'est gratuit à partir de 60 ans, nous ne payons rien; ils ont dit que l'on pouvait se soigner sans débourser de l'argent » (femme, 65 ans). La plupart des personnes âgées témoignaient de la simplicité des procédures pour les consultations : « J'ai montré ma carte d'identité au vigile, il m'a dit que je ne dois pas faire la queue parce que je fais partie des personnes du troisième âge, d'aller montrer ma carte au caissier, lui m'a remis un ticket pour la consultation. Après je suis venue m'asseoir pour attendre mon tour pour voir le médecin et je n'ai rien payé » (femme, 65 ans).

Presque toutes ont aussi vécu l'intermittence de la prise en charge des soins : « De 2006 à 2012, j'allais faire les analyses gratuitement à l'hôpital Principal ou l'hôpital Militaire de Ouakam et puis ça s'est arrêté; ici, de février à octobre, le Plan Sésame n'a plus marché, j'ai dépensé 200 000 XOF durant cette période entre la consultation, les analyses et les médicaments » (femme, 73 ans). Le constat le plus fréquent demeure la prise en charge partielle des dépenses de santé par le Plan Sésame : « Ce n'est pas tout à fait gratuit parce que bien vrai que la consultation est gratuite mais pas les médicaments; la dernière fois que je suis venue ici, ils m'avaient prescrit une ordonnance de 7000 XOF que j'ai achetée dans une pharmacie à l'extérieur » (femme, 66 ans). En effet, le Plan Sésame ne couvre pas l'achat de médicaments de spécialité dont l'achat dans les pharmacies privées est rendu indispensable par les défauts d'approvisionnement en médicaments génériques dans les structures de soins. Un constat similaire est établi pour les analyses biologiques : « Le Plan Sésame prend en charge une partie des analyses; dernièrement, une seule a été couverte par le Plan Sésame. J'ai dépensé 25 000 XOF parce que la radiographie m'a coûté 7000 XOF et j'ai payé 18 000 XOF pour le bilan sanguin, celle que je n'ai pas payée était à 2500 XOF » (femme, 65 ans).

Malgré toutes les limites du dispositif, y compris celles liées à sa mise en œuvre, les personnes âgées témoignaient de l'intérêt qu'elles y trouvaient, tout en considérant que la prise en charge devrait être étendue aux médicaments : « Le Plan Sésame est une bonne chose, mais il faudrait rendre gratuits ou revoir à la baisse les prix des médicaments » (homme, 66 ans); « C'est bien, ça réduit les dépenses partiellement, mais prescrire des médicaments trop chers équivaut à laisser mourir la personne » (homme, 63 ans); « Le Plan Sésame m'aide, oui, mais on ne peut pas s'en tenir qu'à ce qui nous a été donné » (femme, 65 ans).

### Application de la couverture en santé, coûts médicaux et restes à charge

#### Utilisation du dispositif de couverture sanitaire par les personnes âgées

**Figure 1:**
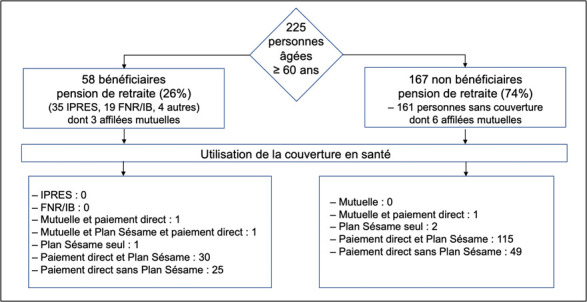
Utilisation du dispositif de couverture en santé par les personnes âgées de 60 ans et plus parmi la population d'enquête (IPRES : Institution de prévoyance retraite du Sénégal; FNR/IB : Fonds national de retraite/ Imputation budgétaire) Use of the health coverage scheme by people aged 60 and over in the study population (IPRES : Institution de prévoyance retraite du Sénégal; FNR/IB : Fonds national de retraite/Imputation budgétaire)

Parmi les 225 personnes âgées enquêtées (Fig. 1), 58 recevaient une pension de retraite (35 versées par l'IPRES, 19 par le FNR, 4 par une autre caisse). Les personnes couvertes par l'IPRES et le FNR auraient donc pu bénéficier de la couverture maladie liée à leur caisse de retraite, 3 personnes étaient aussi affiliées à une mutuelle de santé (communautaire ou professionnelle). Parmi les 165 personnes ne percevant pas de pension de retraite, 6 étaient affiliées à une mutuelle de santé communautaire. Aucune des 225 personnes ne bénéficiait d'une assurance privée en santé.

En pratique, aucune des personnes pouvant utiliser la couverture médicale proposée par l'IPRES et le FNR n'y a eu recours; près de la moitié d'entre elles (33/58) ont eu recours au Plan Sésame en complément d'un paiement direct. Dans l'ensemble, 149 personnes parmi les 225 (66%) ont eu recours au Plan Sésame, 3 (1%) seulement ont utilisé leur mutuelle en complément d'un paiement direct ou en complément du Plan Sésame.

La répartition selon les sexes montre que 71% des hommes ont pu bénéficier d'une couverture médicale contre seulement 63% des femmes. Finalement 74 personnes - le tiers des personnes enquêtées - n'ont bénéficié d'aucune couverture en santé. La solidarité familiale est mobilisée pour 58% des personnes âgées - 67% des femmes, 44% des hommes. En définitive, 7% des personnes interrogées ont déclaré avoir dû repousser la date de leur consultation médicale à cause d'une incapacité financière à supporter les dépenses.

#### Coût médical pour les personnes âgées ayant une HTA et un diabète

Nos observations révèlent que le coût médical moyen de la consultation pour l'hypertension et le diabète sont proches : 32 000 XOF et 37 000 XOF (Tableau II). Comme l'on pouvait s'y attendre, le coût médical moyen pour les personnes souffrant des deux maladies est plus élevé (51 000 XOF); ce renchérissement est dû à la fois aux médicaments et aux examens biologiques. D'une manière générale, la part principale des coûts est liée aux examens biologiques et radiologiques (notamment l'électrocardiogramme, prescrit pour 60 patients; et l'échographique cardiaque, prescrite pour 21 patients); ils constituent de 68% du coût médical moyen pour l'HTA à 75% du coût médical moyen pour le diabète.

**Tableau II:** Coût médical moyen, montant couvert par la CMU, reste à charge médical moyen et coût moyen des frais de déplacement des personnes souffrant d'HTA, de diabète ou des deux pathologies associées, en XOF (francs CFA) Average medical cost, amount covered by the CMU, average out-of-pocket medical expenses and average cost of travel expenses of people suffering from hypertension, diabetes or the two associated pathologies, in XOF (CFA francs)

Pathologies (nombre de personnes)	HTA (46)	Diabète (82)	HTA + diabète (40)
Coût médical moyen *	32 000	37 000	51 000
médicaments	7 000	7 000	12 000
examens biologiques	9 000	23 000	28 000
imagerie et radiologie	13 000	5 000	8 000
Montant moyen couvert par la CMU/Plan Sésame	10 000	16 000	25 000
part prise en charge	31%	43%	49%
Reste à charge médical moyen	21 000	22 000	26 000
médicaments	7 000	7 000	12 000
examens biologiques	5 000	11 000	9 000
imagerie et radiologie	7 000	2 000	3 000
Coût moyen des frais de déplacement	2 000	2 000	2 000

* Les valeurs ont été arrondies au millier le plus proche; elles sont exprimées en XOF (francs CFA)

#### *Montant moyen du reste à charge* médical et du reste à charge total

Bien que le Plan Sésame soit appliqué de manière variable selon les structures, il permet une réduction sensible du montant du reste à charge. Ainsi, le montant moyen couvert par le Plan est de 10 000 XOF pour l'HTA, 16 000 XOF pour le diabète et 25 000 XOF pour l'association des deux maladies. La part prise en charge par le Plan Sésame varie entre 31 et 49%. La prise en charge par le Plan Sésame de la plupart des examens biologiques et radiologiques coûteux influe favorablement sur le montant du reste à charge. Le montant moyen lié aux médicaments, dont aucun n'est couvert par le Plan Sésame, varie de 7 000 XOF à 12 000 XOF; les médicaments constituent entre le tiers et la moitié du reste à charge. Le reste à charge médical moyen est quasi identique pour l'HTA et le diabète (22 000 XOF); il est de 26 000 XOF pour l'association des deux maladies. Le coût moyen des frais de déplacement est de 2 000 XOF, ce montant doit être ajouté au reste à charge médical; ainsi le reste à charge total moyen d'une consultation avec les frais de déplacements pour ces personnes âgées se situe entre 24 000 et 28 000 XOF.

## Discussion

### Forces et limites de l'étude

L'intérêt majeur de cette recherche tient à l'approche empirique mixte - qualitative et quantitative - mise en œuvre pour documenter l'impact du dispositif de couverture maladie sur les dépenses de santé des personnes âgées à Dakar, en se focalisant sur celles atteintes de diabète et/ ou d'hypertension. Cette approche permet de mettre en perspective les perceptions et les pratiques des professionnels de santé et des usagers du dispositif sanitaire, et d'objectiver, par la mesure des coûts et le calcul du reste à charge pour les patients, l'impact sur les dépenses de santé, du point de vue des usagers. La principale limite de cette étude exploratoire tient au choix de deux sites urbains, dans la capitale; les observations et les estimations de dépenses et de restes à charge ne peuvent pas être généralisées à l'ensemble du pays. Étant donné l'extrême variabilité d'application du Plan Sésame selon les structures, il était difficile d'identifier plusieurs structures l'appliquant de manière similaire. Nous avons donc pu calculer les coûts médicaux qui sont indépendants du dispositif de remboursement, mais les restes à charge étaient eux évidemment variables selon l'application du plan Sésame. Cette étude ne présente donc pas de caractère comparatif entre les deux structures, il s'agit d'une approche exploratoire qui pourra être ultérieurement approfondie.

L'hétérogénéité des connaissances des personnes âgées sur la couverture sanitaire

L'enquête met en évidence l'hétérogénéité des connaissances des personnes âgées sur la couverture sanitaire qui leur est destinée, alors même que le Plan Sésame est en place depuis plus de 10 ans. On retrouve un lien étroit entre le niveau d'insertion sociale des personnes et leur utilisation de la couverture sanitaire : les personnes qui sont les mieux insérées dans les dispositifs institutionnels (anciens salariés du secteur formel, cadres de la fonction publique ou du secteur privé) sont les mieux informées et les mieux couvertes; à l'inverse les personnes dont la carrière professionnelle s'est faite dans le secteur informel (ouvriers journaliers, petits commerçants) apparaissent les moins informées et les moins couvertes. C'est notamment le cas pour des hommes et des femmes, anciens commerçants et mères au foyer, dont certains n'ont jamais eu recours au Plan Sésame alors qu'ils ont des dépenses de santé élevées depuis plusieurs années.

Ce processus de sélection des personnes selon leur catégorie socio-économique, qui se traduit par une moindre couverture en santé des plus pauvres, avait déjà été mis en évidence au Sénégal [18, 25], et il a encore été récemment observé au Ghana [6], avec un effet encore plus sensible au détriment des personnes habitant en milieu rural. Parmi les dispositifs de couverture sanitaire, on remarque également un très faible niveau de connaissance sur les mutuelles de santé. Des personnes âgées isolées, ne bénéficiant pas de l'aide d'un proche pouvant leur expliquer le fonctionnement des dispositifs et les accompagner dans les démarches, ne peuvent que s'en remettre au soutien des services sociaux des structures sanitaires, et à défaut ne bénéficient d'aucune gratuité.

Les coûts médicaux de l'HTA et du diabète, la part grandissante de l'imagerie médicale

Les résultats de l'étude des coûts médicaux de l'HTA et du diabète réactualisent les observations réalisées en 2011 par Kâ *et al.* [12], notamment sur la répartition par poste de dépense. En 2011, ces auteurs indiquaient déjà que les examens biologiques et les médicaments de spécialité constituaient les principaux postes de dépenses. Les comparaisons des coûts en valeur absolue avec d'autres pays sont rendues difficiles du fait de modes de calcul différents, mais en proportion, on retrouve bien la part prépondérante de ces deux postes au Mali [4] et dans différents pays d'Afrique subsaharienne [20] pour le diabète, ainsi que pour l'HTA [16, 19]. Dans leur étude réalisée au Mali en 2008-2009, Bermudez *et al.* notaient que les tests de laboratoire représentaient le principal coût pour les personnes atteintes de diabète [4].

Notre étude confirme l'ampleur des coûts liés aux examens complémentaires. Ainsi, à propos du diabète, le bilan biologique minimal recommandé au Sénégal qui devrait être réalisé une fois par an (numération sanguine, glycémie, créatinémie, cholestérol HDL et total, triglycéride, ionogramme, test urine 10 paramètres), auquel les cliniciens ajoutent un électrocardiogramme, est facturé entre

29 000 XOF et 39 000 XOF selon les structures. L'usage de l'échographie cardiaque, facturée 35 000 XOF, commence à se développer. Ces montants accroissent considérablement les coûts de prise en charge. La volonté des cliniciens de fournir un meilleur standard de soins et la disponibilité de ces examens les conduisent à prescrire de plus en plus souvent ces examens coûteux.

Des restes à charge élevés qui accroissent la dépendance des personnes âgées

Notre étude estime le reste à charge lié à une consultation pour les patients âgés présentant une HTA et/ou un diabète entre 24 000 et 28 000 XOF. Ces montants doivent être mis en perspective avec les ressources dont disposent les personnes. Les études statistiques publiées en 2021 [3] rapportent qu'au Sénégal la dépense quotidienne moyenne est de 1390 XOF/personne/jour; et que près de 38% de la population vit avec 913 XOF/ personne/jour, somme qui représente le seuil de pauvreté calculé en 2019. Ainsi, le reste à charge moyen d'une consultation de suivi de l'hypertension, du diabète ou de l'association des deux maladies représente de 15 à 30 jours de dépense quotidienne. Alors que la grande majorité des personnes âgées ne disposent pas de pension de retraite au Sénégal, les dépenses de santé incombent donc à leurs proches. Au sein des ménages, la dépense médicale des personnes âgées entre en concurrence avec les besoins de base, notamment alimentaires, qui captent habituellement plus de la moitié des ressources des ménages. Cet indispensable soutien familial place les personnes âgées en situation de totale dépendance. Or, depuis une vingtaine d'années, diverses études alertent sur l'affaiblissement des solidarités intergénération-nelles mises à l'épreuve du fait de la précarité économique de l'immense majorité des familles [8, 11]. Le contexte de transformation sociale qui modifie les pratiques de solidarité intergénérationnelle rend de plus en plus indispensable le développement d'une solidarité institutionnelle telle que celle proposée par le Plan Sésame.

## Conclusion

Cette étude nous a permis de décrire les perceptions du Plan Sésame par les professionnels de santé et par les personnes âgées, de préciser l'utilisation effective des dispositifs de couverture sanitaire, d'évaluer le montant des dépenses médicales engagées à l'occasion d'une consultation de routine et de calculer les restes à charge liés à une consultation pour des personnes âgées présentant une hypertension artérielle et/ou un diabète.

En 2021, le Plan Sésame ne permet pas encore une totale gratuité des soins pour les personnes âgées. Néanmoins son application, même partielle, se traduit par une réelle baisse des dépenses de santé des personnes âgées. Son utilisation demeure limitée du fait d'une application inconstante par la plupart des structures sanitaires. Son impact est insuffisant au regard des sommes que les usagers doivent débourser dans un contexte de vulnérabilité sociale et économique. Ces observations confortent la nécessité de travailler à la réduction du prix des prestations médicales et au renforcement de la CMU, afin d'améliorer l'équité et la performance du dispositif, et de le rendre entièrement fonctionnel dans toutes les structures de soins.

## Financement

Cette recherche s'inscrit dans le cadre du programme UNISSAHEL (Couverture universelle en santé au Sahel), financé par l'Agence française de développement (AFD).

## Remerciements

Nous remercions les participants à l'étude ainsi que les acteurs de santé du centre Gaspard Kamara et du centre de diabétologie Marc Sankalé de l'hôpital Abass Ndao à Dakar.

## Contributions des auteurs

BT, GLB et KS, ont participé à la conception de la recherche. L'enquête de terrain a été réalisée par BRT, supervisée par BT, GLB et KS. Les analyses ont été réalisées par BT, GLB, KS, BRT et MC. BT et GLB ont rédigé la première version du document, révisé de manière critique et avec l'ajout d'informations substantielles par KS et MC. Tous les auteurs ont approuvé la version finale.

## Liens d'intérêts

Les auteurs ne déclarent aucun conflit d'intérêts.

## Déclaration éthique

L'enquête sur le dispositif de protection sociale et les restes à charge entre dans la composante #2 « Anthropologie et approches micro-sociales qualitatives » du programme UNISSAHEL approuvé par le Comité national d'éthique pour la recherche en santé du Sénégal (n° 000053/MSAS/DPRS/CNERS et n° 0000118/MSAS/DPRS/CNERS).

## Disponibilité des données

Les données sont disponibles sur demande argumentée auprès de l'auteur correspondant, et après accord des investigateurs principaux de la recherche.
